# CCK-sensitive C fibers activate NTS leptin receptor-expressing neurons via NMDA receptors

**DOI:** 10.1152/ajpregu.00238.2022

**Published:** 2023-12-18

**Authors:** Drew M. Neyens, Lynne Brenner, Rowan Calkins, Eric T. Winzenried, Robert C. Ritter, Suzanne M. Appleyard

**Affiliations:** Department of Integrated Physiology and Neuroscience, Washington State University, Pullman, Washington, United States

**Keywords:** cholecystokinin, leptin, leptin receptor, nucleus of the solitary tract, vagus

## Abstract

The hormone leptin reduces food intake through actions in the peripheral and central nervous systems, including in the hindbrain nucleus of the solitary tract (NTS). The NTS receives viscerosensory information via vagal afferents, including information from the gastrointestinal tract, which is then relayed to other central nervous system (CNS) sites critical for control of food intake. Leptin receptors (lepRs) are expressed by a subpopulation of NTS neurons, and knockdown of these receptors increases both food intake and body weight. Recently, we demonstrated that leptin increases vagal activation of lepR-expressing neurons via increased NMDA receptor (NMDAR) currents, thereby potentiating vagally evoked firing. Furthermore, chemogenetic activation of these neurons was recently shown to inhibit food intake. However, the vagal inputs these neurons receive had not been characterized. Here we performed whole cell recordings in brain slices taken from lepRCre × floxedTdTomato mice and found that lepR neurons of the NTS are directly activated by monosynaptic inputs from C-type afferents sensitive to the transient receptor potential vanilloid type 1 (TRPV1) agonist capsaicin. CCK administered onto NTS slices stimulated spontaneous glutamate release onto lepR neurons and induced action potential firing, an effect mediated by CCKR_1_. Interestingly, NMDAR activation contributed to the current carried by spontaneous excitatory postsynaptic currents (EPSCs) and enhanced CCK-induced firing. Peripheral CCK also increased c-fos expression in these neurons, suggesting they are activated by CCK-sensitive vagal afferents in vivo. Our results indicate that the majority of NTS lepR neurons receive direct inputs from CCK-sensitive C vagal-type afferents, with both peripheral and central CCK capable of activating these neurons and NMDARs able to potentiate these effects.

## INTRODUCTION

Obesity results from a prolonged discrepancy between energy expenditure and caloric intake, such that an individual consumes more frequent and/or larger meals than are necessary to meet the body’s metabolic demands (1). Importantly, feeding behavior is controlled by the nervous system, and the neural determinants of meal size are informed by satiety signals originating from the gastrointestinal (GI) tract (2–4). The nucleus of the solitary tract (NTS) in the brain stem receives signals directly from the GI via the vagus nerve, and this information is then relayed to other brain regions involved in the process of satiation and meal termination (5–7). Moreover, the NTS also receives direct inputs from the area postrema (8) as well as other brain regions involved in the control of food intake (7) and contains fenestrated capillaries, which may allow some NTS neurons to respond directly to humoral signals (9). Thus, the NTS is ideally positioned to integrate neural and humoral satiety signals in the control of food intake.

Leptin is an anorexigenic hormone critical for normal energy homeostasis (10, 11). Leptin inhibits food intake largely through its actions in the central nervous system (CNS) (12). Studies of leptin have historically focused on anorexigenic effects exerted in the hypothalamus and, more recently, in regions involved in reward. Nevertheless, there is substantial evidence indicating that leptin also increases the intake-suppressive effects of vagally mediated satiation signals in the hindbrain (13–17). Moreover, knockdown of leptin receptors (lepRs) in hindbrain NTS neurons induces hyperphagia and attenuates the effectiveness of GI signals to reduce food intake (18–20), demonstrating that endogenous lepR signaling in the NTS is required for normal control of food intake. Our laboratory recently has shown that lepR neurons in the NTS are both directly and indirectly activated by stimulation of vagal afferents in the solitary tract (ST) and that leptin increases synaptic throughput at the ST-NTS synapse (21). In addition, others have reported that in vivo activation of lepR neurons results in inhibition of food intake (22). However, the specific types of vagal afferent fibers that activate these critical lepR neurons remain unknown.

The NTS receives input from both myelinated A-type and unmyelinated C-type vagal afferents from the GI tract, which release glutamate as their primary neurotransmitter, therewith activating both AMPA-type (AMPARs) and NMDA-type (NMDARs) glutamate receptors in second-order NTS neurons (23–26). The majority of vagal afferents carrying GI information are C fibers that express the transient receptor potential vanilloid type 1 (TRPV1) ion channel (27), whereas most other vagal afferent fiber types, e.g., A and Aδ fibers, do not express TRPV1 (28, 29).

Cholecystokinin (CCK) is a gut peptide that reduces food intake, typically through reductions in meal size (30–32). Importantly, the effects of peripheral CCK are largely mediated by the vagus nerve (33–36), and most CCK-sensitive vagal afferents are C fibers that are sensitive to capsaicin (27, 37, 38). Interestingly, NTS NMDARs are critical for peripheral CCK-induced reductions in food intake (39, 40), suggesting that this effect depends on activation of NTS NMDARs by capsaicin-sensitive C fibers.

We have recently reported that NTS lepR neurons exhibit larger ST-evoked NMDAR currents than most other NTS neurons (21), suggesting that NMDARs are particularly important in how these cells process excitatory information from the solitary tract. Indeed, NMDAR antagonists block the acute effects of leptin on synaptic throughput as well as attenuate NTS leptin-induced reductions in food intake (21). However, the role of NMDARs in mediating CCK activation of NTS lepR neurons at the synaptic level is not known.

In the present study, we determined that NTS lepR neurons receive monosynaptic input from C-type vagal afferent fibers that are sensitive to CCK. We also show that CCK-induced glutamate release from vagal afferents drives action potential (AP) firing in lepR neurons through vagal afferent CCK_1_ receptors and that NMDARs increase the sensitivity of NTS neurons to this excitatory effect of CCK. Finally, we confirm that peripheral CCK increases c-fos expression in a subpopulation of lepR neurons in vivo.

## MATERIALS AND METHODS

### Animals

All mice were maintained on a 12:12-h light-dark cycle at room temperature in the Department of Integrative Physiology and Neuroscience vivarium and had ad libitum access to standard chow and water except during overnight fasts, as described below. Transgenic mice were bred by crossing leptin receptor-Cre animals (LepR-Cre; Martin Myers) with a fluorescent reporter mouse line (Lox-P-Rosa tdTomato; Allen Brain Institute); animals were maintained on a C57BL/6J background. A total of 78 mice (41 males and 37 females) were used for electrophysiological studies, and 12 mice (5 males and 7 females) were used for immunohistochemical experiments. All animal procedures were conducted with the approval of the Institutional Animal Care and Use Committees at Washington State University (WSU) and in accordance with the US Public Health Service Policy on Humane Care and Use of Laboratory Animals (PHS Policy) and the National Institutes of Health *Guide for the Care and Use of Laboratory Animals* (NIH Guide).

### Horizontal NTS Slices

Approximately equal numbers of adult male and female transgenic mice (8–16 wk) were used in all electrophysiological experiments. Mice were deeply anesthetized with isoflurane and then euthanized by creation of an acute pneumothorax (thoracic compression). The hindbrain was rapidly removed and placed for 1 min in cold (0–4°C) artificial cerebrospinal fluid (aCSF) composed of the following (in mM): 125 NaCl, 3 KCl, 1.2 KH_2_PO_4_, 1.2 MgSO_4_, 25 NaHCO_3_, 2 CaCl_2_, and 10 dextrose, bubbled with 95% O_2_-5% CO_2_. For Mg^2+^-free experiments, MgSO_4_ was omitted from the aCSF used for recording. The final osmolarity for all aCSF solutions was adjusted to 305–307 mosmol/L with sucrose. The medulla was trimmed and a wedge removed from the ventral surface to align the solitary tract (ST) with the NTS in the same cutting plane when mounted in a vibrating microtome (Leica VT-1000S). Hindbrain slices (250 µm) were cut with a sapphire knife (Delaware Diamond Knives, Wilmington, DE) and contained a long section of the ST. Slices were submerged in a perfusion chamber and allowed to recover for 1 h in oxygenated aCSF flowing at a rate of ∼2 mL/min. All recordings were performed at a near-constant temperature (31.5–32°C) and pH 7.4.

### Whole Cell Electrophysiology

LepRCre-tdTomato neurons were visually selected for recordings with a fluorescence microscope (Olympus BX51WI), and recording electrodes were guided to neurons one to three cell layers deep with differential interference contrast (DIC) optics. NTS neurons were recorded within 200 µm rostral or caudal of the obex and medial to the ST. Recording electrodes (3.2–3.8 MΩ) were filled with internal solution composed of the following (in mM): 10 NaCl, 130 potassium gluconate, 11 EGTA, 1 CaCl_2_, 2 MgCl_2_, 10 HEPES, 2 NaATP, and 0.2 NaGTP, pH 7.3, 297–300 mosmol/L. Whole cell voltage- and current-clamp recordings were made with an Axopatch 700B amplifier, a Digidata 1440A digitizer, and pCLAMP 10 software (all from Molecular Devices, Sunnydale, CA). Solutions flowing to the recording chamber were heated with a HPRE2 preheater (Cell MicroControls, Norfolk, VA), and bath temperature was monitored through a probe positioned next to the slice. Neurons with holding currents exceeding ±100 pA at holding voltage (*V*_h_) = −60 mV during the initial 10-min control period were excluded from further study. For current-clamp (CC) experiments, only neurons with all action potentials reaching above 0 mV were included in the study. Series resistance was monitored throughout recordings, and neurons were excluded from analysis if it exceeded 20 MΩ or varied >5 MΩ. Synaptic responses were evoked with an ultrafine concentric bipolar stimulating electrode [50-µm inner diameter (ID); FHC, Inc., Bowdoin, ME] placed on the ST 1–2 mm from the recording electrode. Electrical stimuli were delivered from an isolated programmable stimulator (Isoflex stimulator with Master-8; AMPI, Jerusalem, Israel) triggered to deliver a burst of stimuli (5–50 Hz). Neurons were only included if electrical stimulation of the ST recruited one or more afferents contributing to an excitatory postsynaptic current (EPSC).

### Immunohistochemistry

Six- to twelve-week-old lepRCre × floxed tdTomato mice were fasted overnight (16 h) before the experiment to isolate the effects of exogenous CCK from those resulting from ingestion of food. On the morning of the experiment, mice received an intraperitoneal injection of either volume-matched 0.9% sterile NaCl or CCK (5 µg/kg or 10 µg/kg doses in 0.9% NaCl). Ninety minutes after the injection, mice were rapidly anesthetized with isoflurane anesthetic (Patterson Veterinary) and perfused intracardially with 0.1 M phosphate-buffered saline (pH 7.4) followed by 4% paraformaldehyde (Electron Microscopy Sciences) in 0.1 M phosphate buffer (PB), pH 7.4. Brains were immediately removed, postfixed in the same fixative for 2 h, and cryoprotected in 25% sucrose-0.1 M PB solution overnight at 4°C. Coronal cryostat sections cut at 30 µm were collected for immunostaining. Tissue sections were blocked with 10% normal horse serum in TPBS with 0.3% Triton X-100 at room temperature for 1 h. Sections were incubated with goat anti-c-fos (sc-52-G, 1:1,000; Santa Cruz Biotechnology), an antibody that has been used for both rats and mice (41–46), for 48 h at 4°C with subsequent incubation in Alexa Fluor 488 donkey anti-goat (1:1,000; Life Technologies). Stained sections were mounted on slides and coverslipped with ProLong Gold Antifade reagent with DAPI (Invitrogen). Digital images were captured at ×20 and stitched with Nikon Elements image-processing software. The total number of cell bodies containing native tdTomato fluorescence and c-fos immunoreactivity were counted in the NTS in four equivalent sections at rostrocaudal levels: 7.92, 7.64, 7.48, and 7.20 mm caudal to bregma (47), labeled respectively as NTS *levels 1*, *2*, *3*, and *4* in this study. The numbers of *c-fos*-immunoreactive (IR) cells are presented as averages for each rostrocaudal level, and colocalization is presented as the percentage of *c-fos* IR cells that contain tdTomato fluorescence and vice versa.

### Drugs

(*E*)-*N*-[(4-hydroxy-3-methoxyphenyl)methyl]-8-methyl-6-nonenamide (capsaicin), D-4-[(2*E*)-3-phosphono-2-propenyl]-2-piperazinecarboxylic acid (DCP), 2,3-dioxo-6-nitro-1,2,3,4-tetrahydrobenzo[f]quinoxaline-7-sulfonamide (NBQX), 4-[[(1*R*)-2-[[(2*R*)-3-(1*H*-indol-3-yl)-2-methyl-1-oxo-2-[[(tricyclo[3.3.1.1^3,7^]dec-2-yloxy)carbonyl]amino]propyl]amino]-1-phenylethyl]amino]-4-oxobutanoic acid (CI988, CCKR_2_ antagonist), cholecystokinin (CCK octapeptide, sulfated), and serotonin hydrochloride (5-HT) were obtained from Tocris Cookson (Minneapolis, MN). (±)-4-[(3,4-Dichlorobenzoyl)amino]-5-(dipentylamino)-5-oxopentanoic acid (lorglumide, CCKR_1_ antagonist) was obtained from Sigma-Aldrich (St. Louis, MO).

### Data Analysis

The frequency of spontaneous excitatory postsynaptic currents (sEPSCs), spontaneous excitatory postsynaptic potentials (sEPSPs), action potentials (Hz), and the amplitude of inward current in voltage clamp (pA) or membrane depolarization in current clamp (mV) were analyzed with Mini Analysis 6.0.3 (Synaptosoft, Decatur, GA) and Clampfit 10.3 (Molecular Devices) software. All data are presented as means ± SE. Both male and female animals were used for electrophysiological experiments, with a roughly equal distribution of male and female mice for all conditions. We found no significant differences in responses between male and female mice; therefore, data were combined for further analysis and *n* refers to the numbers of independent cells recorded per condition or the number of animals for immunohistochemical experiments. For capsaicin and CCK treatments, sEPSC amplitude and frequency were measured during a 30-s window centered on the peak effect (∼2–2.5 min) and compared with the last 3 min of aCSF. 5-HT had a slower effect; therefore, sEPSC frequency and amplitude were measured as the average of *minutes 2–5* of 5-HT application compared with the last 5 min of the aCSF control period. Kolmogorov–Smirnov (K-S tests) on these time frames were used to determine whether changes in sEPSC or sEPSP frequency were significant within individual neurons, and further statistical comparisons were made between neurons with Mann–Whitney, Student’s *t* test, one-way ANOVA, Friedman, Kruskal–Wallis, and two-way ANOVA, with post hoc Bonferroni or Šídák’s multiple comparison analysis where appropriate (SigmaPlot 14.0, GraphPad Prism 9). Differences were considered statistically significant for *P* values < 0.05.

## RESULTS

### NTS lepR Neurons Receive Input from C-Type Afferent Fibers

**Figure 1.**
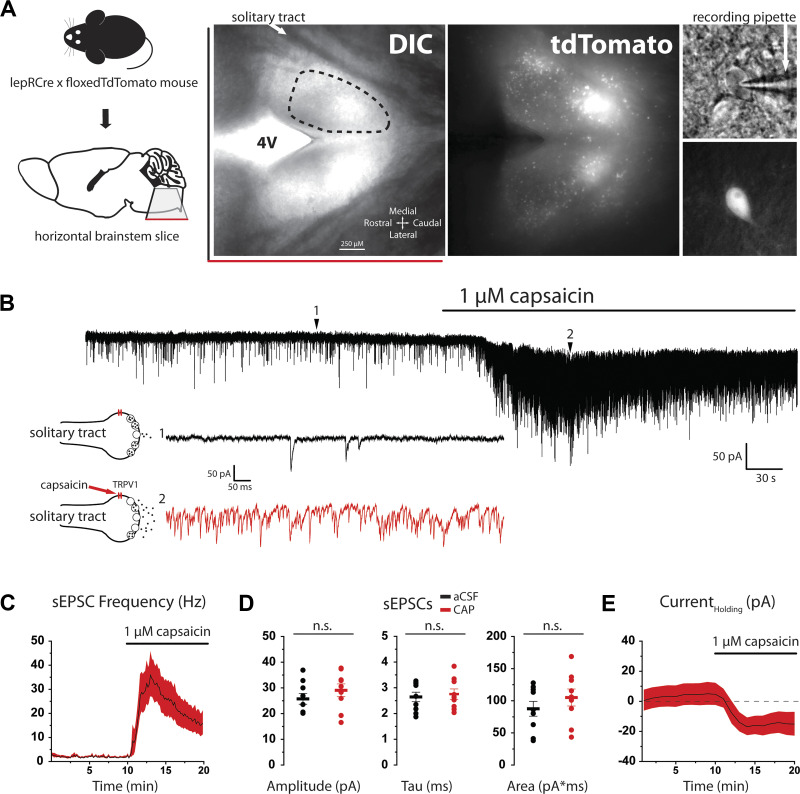
Capsaicin stimulates spontaneous glutamate release onto nucleus of the solitary tract (NTS) leptin receptor-expressing (lepR) neurons. *A*: horizontal brain slice taken from lepR-Cre × floxedTdTomato mouse including the solitary tract (arrow), 4th ventricle (4V), and NTS (dotted region). Neurons were recorded from both the left and right NTS. lepR neurons were identified by tdTomato fluorescence, and recording pipettes were guided to cells with differential interference contrast (DIC; *right*). *B*: voltage-clamp [holding voltage (*V*_h_) = −60 mV] trace of lepR neuron showing that capsaicin increases spontaneous excitatory postsynaptic current (sEPSC) frequency and induces an inward current [*inset*: 1-s traces showing sEPSCs before capsaicin (*1*, black) and after capsaicin (*2*, red)]. TRPV1, transient receptor potential vanilloid type 1. *C*: time course of sEPSC frequency during capsaicin application. sEPSC frequency was averaged in 10-s bins and displayed as the average across cells (black line) ± SE (shaded region). *D*: graphs showing distribution and mean (± SE) of sEPSC current amplitudes, decay time constants (tau), and area before (black) and after (red) capsaicin (CAP). aCSF, artificial cerebrospinal fluid. n.s., Not significant. *E*: time course of holding current (Current_Holding_) during capsaicin application. Holding current was averaged in 1-min bins and displayed as the average across cells (black line) ± SE (shaded region).

Horizontal brain stem slices containing the ST and NTS were obtained from lepRCre × floxed tdTomato animals, and the cell bodies of tdTomato-positive neurons were easily identified for recordings by epifluorescence illumination under high magnification as previously described (Fig. 1*A*).

We have previously shown that NTS lepR neurons receive both monosynaptic and polysynaptic (direct and indirect) excitatory inputs after ST stimulation (21). However, the type of afferent inputs was not known. To determine whether lepR neurons receive input from C-type fibers, we performed voltage-clamp recordings and measured changes in spontaneous EPSCs (sEPSCs) in response to the potent TRPV1 agonist capsaicin. Activation of TRPV1 on vagal afferent terminals increases spontaneous glutamate release onto NTS neurons (48), and this is an effective method for indicating synaptic input from TRPV1-expressing C fibers. Bath application of 1 µM capsaicin dramatically increased sEPSC frequency in all lepR neurons recorded [*F*(119) = 6.78, *P* < 0.001, 1-way ANOVA, *n* = 9; Fig. 1, *B* and *C*]. This effect was maximal 3 min into capsaicin treatment (35.9 ± 9.1 Hz), after which time sEPSC frequency began to decline but did not reach baseline levels. Figure 1*D* shows that there were no significant differences in sEPSC amplitude (25.7 ± 2.1 vs. 29.1 ± 2.5 pA; *P* = 0.318), tau (2.645 ± 0.186 vs. 2.756 ± 0.201 ms; *P* = 0.691), or area (87.6 ± 11.6 vs. 105 ± 13.2 pA·ms; *P* = 0.333) after capsaicin treatment. A change in frequency but not amplitude or decay time is consistent with a presynaptic action of capsaicin to increase glutamate release. We did observe a significant shift in holding current over time in the presence of capsaicin [χ^2^(19, *n* = 9) = 59.73, *P* < 0.001, Friedman ANOVA; Fig. 1*E*]; however, the onset and peak of this shift in holding current were closely timed with the massive spike in glutamate release. Work from others has shown that capsaicin induces a change in holding current in some NTS neurons that is blocked by glutamate receptor antagonists (49).

### NTS lepR Neurons Receive Monosynaptic Input from C-Type Afferents and Polysynaptic Input from TRPV1-Negative Afferents

**Figure 2.**
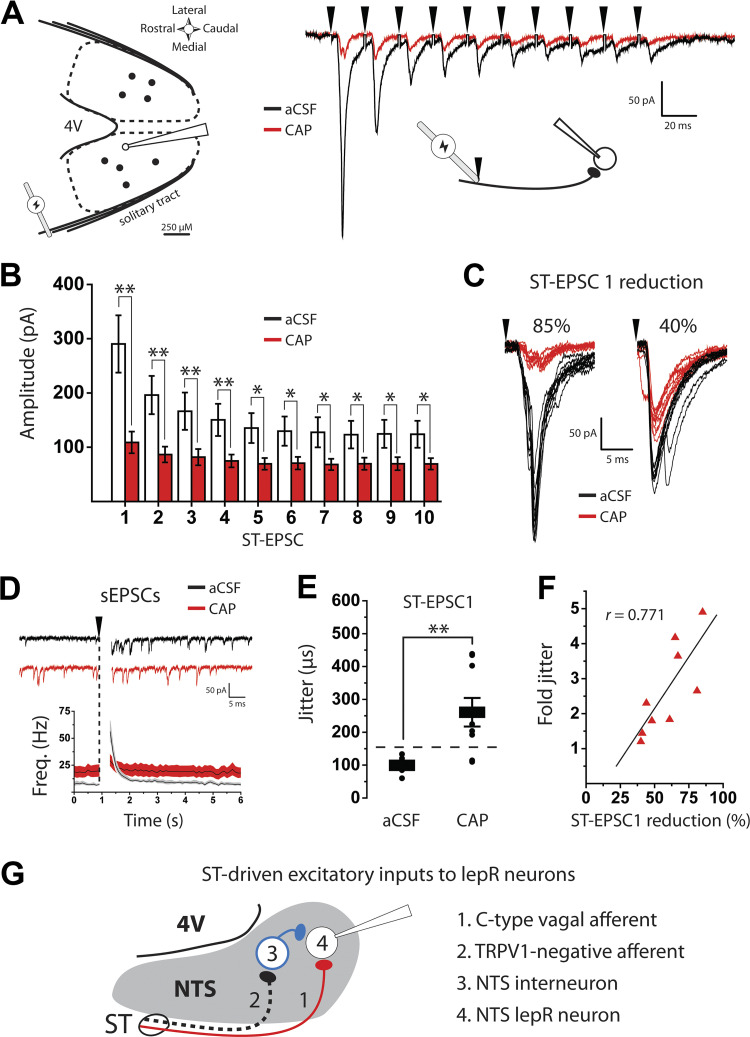
Nucleus of the solitary tract (NTS) leptin receptor-expressing (lepR) neurons receive monosynaptic input from capsaicin-sensitive fibers and polysynaptic input from transient receptor potential vanilloid type 1 (TRPV1)-negative fibers. *A*, *left*: approximate distribution of recorded lepR neurons within the NTS and placement of stimulating electrode on the solitary tract. 4V, 4th ventricle. *Right*: voltage-clamp [holding voltage (*V*_h_) = −60 mV] traces of a lepR neuron showing that 10 stimulations of the solitary tract (ST, black arrows) at 50 Hz produces ST-evoked excitatory postsynaptic currents (ST-EPSCs). ST-EPSCs are displayed as the average of 30 sweeps before (black) and after (red) capsaicin (CAP). aCSF, artificial cerebrospinal fluid. *B*: graph showing mean (±SE) ST-EPSC current amplitudes during the stimulus train before (black) and after (red) capsaicin. *C*: voltage-clamp traces showing the capsaicin-induced reduction of ST-EPSC 1 in 2 different cells. ST-EPSCs are displayed as 10 overlapping sweeps before (black) and after (red) capsaicin. *D*, *top*: example voltage-clamp traces showing sEPSCs following stimulation of the solitary tract before (black) and after (red) capsaicin. *Bottom*: time course of sEPSC frequency displayed as the average across cells (black lines) ± SE (shaded regions). *E*: graph showing distribution and mean (± SE) of ST-EPSC 1 jitter before and after capsaicin. *F*: scatterplot and linear regression of fold increase in jitter vs. % ST-EPSC 1 reduction after capsaicin (*r* = Pearson correlation coefficient). *G*: proposed diagram of ST-driven excitatory inputs to NTS lepR neurons. C-type afferent fibers (*1*, solid red) synapse directly onto NTS lepR neurons (*4*), whereas TRPV1-negative afferents (*2*, dashed black line) synapse onto unidentified glutamatergic interneurons (*3*, blue). The presence of any putative TRPV1-expressing nonvagal fibers is omitted for clarity. *P < 0.05, **P < 0.001.

Next, we determined whether monosynaptic ST-EPSCs were mediated by C fibers. Prolonged TRPV1 activation leads to significant depletion of glutamate from afferent terminals and attenuates ST-evoked C fiber-mediated EPSCs (28, 29, 49). We used this approach to determine the extent to which the lepR neurons receive C fiber inputs. lepR neurons were recorded throughout the medial NTS, and stimulation of the solitary tract produced ST-EPSCs in all neurons recorded (*n* = 9; Fig. 2*A*). Before capsaicin, 10 ST shocks delivered at 50 Hz produced ST-EPSCs that displayed frequency-dependent depression (FDD) characteristic of monosynaptic, high probability of release synapses. After 10 min of capsaicin, ST-EPSC amplitudes were significantly reduced during the stimulus train in all cells [*F*(1,9) = 14.6, *P* < 0.001, 2-way ANOVA], and the remaining capsaicin-insensitive EPSCs did not exhibit the same robust FDD (Fig. 2*B*). This capsaicin-induced reduction in ST-EPSC amplitude was greatest during the first stimulation (ST-EPSC1: 290.2 ± 46.5 vs. 108.8 ± 17.6 pA, *P* = 0.002, paired *t* test; Fig. 2*B*), and ST-EPSC1 reduction ranged from 85% to 40% within individual cells (respective examples; Fig. 2*C*). These data suggest that the majority of the afferent inputs to lepR neurons are from capsaicin-sensitive C fibers. Interestingly, smaller capsaicin-insensitive ST-EPSCs remained in most lepR neurons.

Another hallmark of TRPV1-expressing C fibers is asynchronous glutamate release following ST stimulation (48). To measure asynchronous release, sEPSC frequency was averaged in 100-ms bins in the time preceding each ST stimulation and again after the 10th ST-EPSC. sEPSC frequency was significantly larger across all lepR neurons in the 100-ms period following ST stimulation (7.0 ± 1.0 vs. 56.0 ± 10.2 Hz, *P* < 0.001, *n* = 9, Mann–Whitney; Fig. 2*D*), after which time sEPSC frequency quickly returned to baseline. Similar to the data shown in Fig. 1, capsaicin significantly increased baseline sEPSC frequency [*F*(1,8) = 6.24, *P* = 0.037, *n* = 9, 2-way ANOVA; Fig. 2*D*]. However, asynchronous release was diminished after capsaicin, and there was no significant difference in sEPSC frequency before and after ST stimulation (19.07 ± 5.08 vs. 29.0 ± 5.44, *P* = 0.2, Student’s *t* test; Fig. 2*D*).

Standard deviation (SD) of ST-EPSC latency (or “jitter”) can be used to identify monosynaptic inputs to the solitary tract versus polysynaptic pathways, with a SD < 200 µs indicating monosynaptic connections and SD > 200 µs indicating polysynaptic connections (50). Interestingly, we observed a significant increase in jitter in the first ST-EPSC when comparing before and after capsaicin treatment (95.9 ± 7.9 vs. 265.0 ± 49.1 µs, *P* = 0.004, Student’s *t* test; Fig. 2*E*), suggesting that the capsaicin-insensitive inputs (i.e., non-C fibers) were likely polysynaptic. Furthermore, there was a significant correlation between the fold increase in jitter and percent reduction of ST-EPSC1 amplitudes by capsaicin (*r* = 0.771, *P* = 0.025, Pearson correlation; Fig. 2*F*). This is to say that as ST-EPSC amplitude decreased in the presence of capsaicin, the remaining compound EPSC was mediated more by underlying polysynaptic inputs. Together, the data from Figs. 1 and 2 suggest that NTS lepR neurons receive direct input from C-type afferent fibers in addition to polysynaptic input from TRPV1-negative fibers (Fig. 2*G*).

### Most NTS lepR Neurons Receive Input from CCK-Sensitive but Not Serotonin-Sensitive Fibers

**Figure 3.**
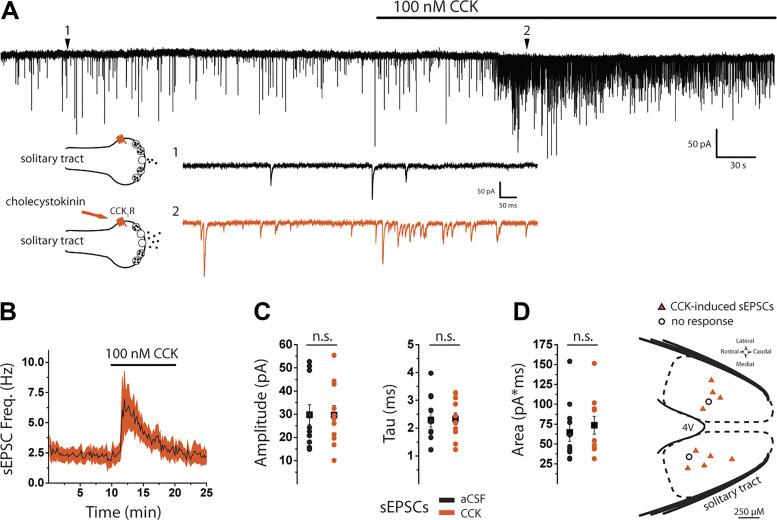
Cholecystokinin (CCK) stimulates spontaneous glutamate release onto nucleus of the solitary tract (NTS) leptin receptor-expressing (lepR) neurons. *A*: voltage-clamp [holding voltage (*V*_h_) = −60 mV] trace of lepR neuron showing that CCK increases spontaneous excitatory postsynaptic current (sEPSC) frequency [*inset*: 1-s traces showing sEPSCs before (*1*, black) and after (*2*, orange) CCK]. CCK_1_R, CCK_1_ receptor. *B*: time course of sEPSC frequency during CCK application. sEPSC frequency was averaged in 10-s bins and displayed as the average across cells (black line) ± SE (shaded region). *C*: graphs showing distribution and mean (± SE) of sEPSC current amplitudes, tau, and area before (black) and after (orange) CCK. aCSF, artificial cerebrospinal fluid. n.s., Not significant. *D*: distribution of recorded lepR neurons within the NTS showing CCK-induced sEPSCs (orange triangles, *n* = 9) and nonresponders (open circles, *n* = 2).

The majority of C-type vagal afferent fibers carrying information from the stomach and duodenum express the cholecystokinin A receptor (CCKR_1_) and are activated by CCK (27), and activation of CCKR_1_ on afferent terminals stimulates glutamate release onto NTS neurons (25, 51–53). To determine whether NTS lepR neurons receive input from CCK-sensitive fibers, we applied CCK and examined sEPSCs. Before sEPSCs were recorded, the same stimulus train described in Fig. 2 was briefly applied to confirm that each cell was connected to the solitary tract. Bath application of 100 nM CCK significantly increased sEPSC frequency in 9 out of 11 neurons [χ^2^(145, *n* = 11) = 535.7, *P* < 0.001, Friedman ANOVA; Fig. 3, *A* and *B*]. sEPSC frequency peaked ∼2 min into CCK treatment (7.01 ± 2.03 Hz), declined over the next 8 min, and was comparable to baseline levels by the end of a 5-min wash (2.15 ± 0.61 vs. 2.33 ± 0.68 Hz, *P* = 0.896, Mann–Whitney; Fig. 3*B*). When comparing aCSF and CCK treatment periods, we did not observe significant changes in sEPSC amplitude (29.76 ± 4.2 vs. 29.69 ± 4.04 pA, *P* = 0.991), tau (2.297 ± 0.26 vs. 2.33 ± 0.215 ms, *P* = 0.922), or area (64.60 ± 11.20 vs. 73.69 ± 11.17 pA·ms, *P* = 0.431; Fig. 3*C*). Moreover, we did not observe a significant change in holding current over time [χ^2^(19, *n* = 11) = 26.48, *P* = 0.117, Friedman ANOVA]. A map of recorded lepR neurons did not reveal an obvious pattern between CCK-induced increases in sEPSC frequency and location within the NTS (Fig. 3*D*).

In contrast to the high rate of response to CCK, 100 nM 5-HT did not significantly increase sEPSC frequency compared with aCSF on average [aCSF 1.37 ± 0.38 vs. 5-HT 1.65 ± 0.49 Hz, *n* = 25, *P* > 0.05, 2-way repeated-measures (RM) ANOVA]. When individual lepR neuron responsiveness via K-S tests was compared, 5-HT significantly increased sEPSC frequency in 5 of 25 lepR neurons examined (aCSF 1.35 ± 0.78 vs. 5-HT 3.04 ± 1.49 Hz, *P* < 0.05, 2-way RM ANOVA), significantly decreased frequency in 4 neurons (aCSF 1.82 ± 0.62 vs. 5-HT 1.19 ± 0.41 Hz, *P* < 0.05, 2-way RM ANOVA), and did not significantly change sEPSC frequency in 15 neurons. sEPSC amplitude also did not change uniformly across all cells (aCSF 23.95 ± 1.14 vs. 5-HT 23.42 ± 1.29 pA, *n* = 25, *P* < 0.05, 2-way RM ANOVA). When individual responsiveness was compared, sEPSC amplitude was significantly increased in three neurons (aCSF 24.99 ± 5.10 vs. 5-HT 30.61 ± 7.45 pA, *P* < 0.05, 2-way RM ANOVA) and decreased in five (aCSF 24.79 ± 1.99 vs. 5-HT 20.70 ± 2.04 pA, *P* < 0.05, 2-way RM ANOVA). Changes in sEPSC frequency did not correlate with changes in sEPSC amplitude. Interestingly, we did see some changes in baseline in some cells (13 increased and 5 decreased), suggesting that serotonin may have direct postsynaptic effects in a subset of lepR neurons. These effects did not correlate with changes in sEPSC frequency or amplitude.

### CCK Stimulates Action Potential Firing in NTS lepR Neurons

**Figure 4.**
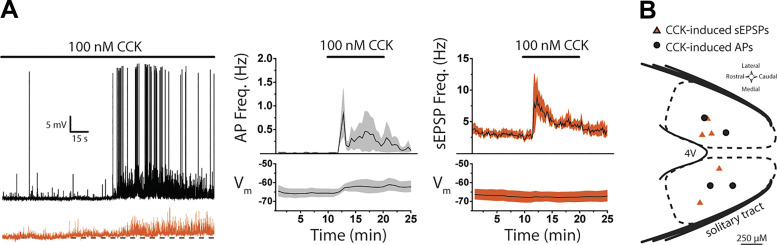
CCK stimulates action potential firing in nucleus of the solitary tract (NTS) leptin receptor-expressing (lepR) neurons. A, *left*: current-clamp traces showing CCK-induced action potential (AP) firing (black) or spontaneous excitatory postsynaptic potentials (sEPSPs, orange). *Right*: time course of membrane potential (*V*_m_), AP frequency, or sEPSP frequency during CCK application displayed as the average across cells (black lines) ± SE (shaded regions). *B*: distribution of recorded lepR neurons within the NTS showing CCK-induced APs (black circles, *n* = 4) or sEPSPs (orange triangles, *n* = 5). DCP, D-4-[(2*E*)-3-phosphono-2-propenyl]-2-piperazine carboxylic acid; n.s., Not significant.

Next, we performed current-clamp (CC) recordings to examine whether CCK-induced glutamate release is sufficient to drive action potential firing. Connection to the solitary tract was confirmed in voltage clamp as before, after which lepR neurons were allowed several minutes to acclimate to CC conditions and reach a stable membrane potential (*V*_m_). On average, lepR neurons rested at a relatively hyperpolarized potential (*V*_m_ = −66.6 ± 1.74 mV; *n* = 9) and rarely fired action potentials during a 10-min control period. In four of nine neurons, bath application of 100 nM CCK induced a spike in action potential firing after 2 min (0.8 ± 0.5 Hz), and firing remained slightly elevated but variable for the remainder of the recording (Fig. 4*A*). This spike in firing was accompanied by a small membrane depolarization (3.7 ± 1.4 mV) that remained significantly elevated into the wash period [*F*(24) = 5.74, *P* < 0.001, 1-way ANOVA; Fig. 4*A*]. In the five neurons that did not fire action potentials, CCK increased the frequency of spontaneous excitatory postsynaptic potentials (sEPSPs) [χ^2^(149, *n* = 5) = 229.46, *P* < 0.001, Friedman ANOVA; Fig. 4*A*]. sEPSP frequency peaked 2 min into CCK treatment (9.0 ± 2.9 Hz) and then declined but did not reach baseline levels (Fig. 4*A*). Membrane potential did not shift in these five cells [*F*(24) = 1.06, *P* = 0.406, 1-way ANOVA; Fig. 4*A*]. Direct current injections were applied after the wash period to confirm that these cells were still capable of firing action potentials. A map of recorded lepR neurons shows that all cells (*n* = 9) responded to CCK with either increased AP firing or sEPSP frequency, but there were no obvious patterns in the distribution of responses (Fig. 4*A*). Taken together with data from Fig. 3, these data suggest that the vast majority of NTS lepR neurons receive input from CCK-sensitive fibers and CCK-induced glutamate release is sufficient to stimulate action potential firing in some lepR neurons.

### NMDARs Contribute to sEPSC Charge Transfer

**Figure 5.**
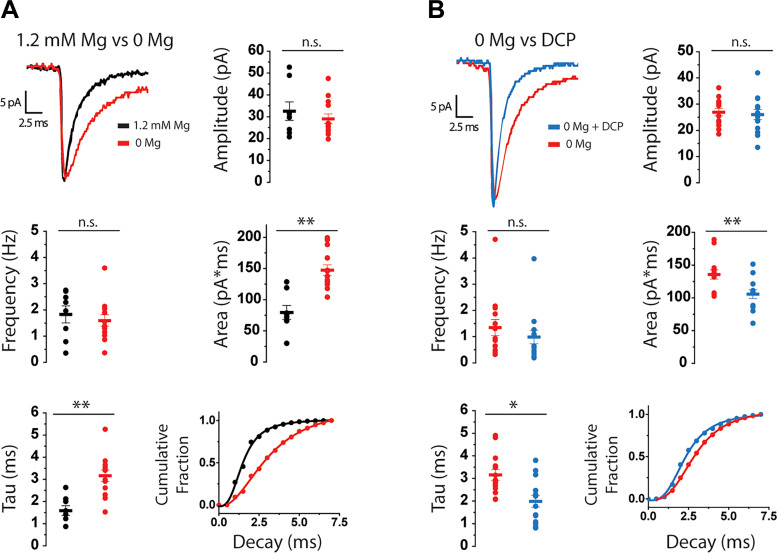
NMDA receptors (NMDARs) contribute to spontaneous excitatory postsynaptic current (sEPSC) charge transfer in nucleus of the solitary tract (NTS) leptin receptor-expressing (lepR) neurons. *A*, *top left*: averaged sEPSC traces from lepR neurons recorded in 1.2 mM Mg^2+^ concentration ([Mg^2+^]) artificial cerebrospinal fluid (aCSF) (black) and in 0 [Mg^2+^] aCSF (red). Example traces were averaged from >100 sEPSCs. Graphs show distribution and mean (±SE) of sEPSC current amplitudes, frequencies, tau, and area. *Bottom right*: cumulative fraction plot of sEPSC decay time. *B, top left*: averaged sEPSC traces from a lepR neuron recorded in 0 [Mg^2+^] aCSF before (red) and after (blue) application of 10 µM D-4-[(2*E*)-3-phosphono-2-propenyl]-2-piperazine carboxylic acid (DCP). Example traces were averaged from >100 sEPSCs. Graphs show distribution and mean (±SE) of sEPSC current amplitudes, frequencies, tau, and area. *Bottom right*: cumulative fraction plot of sEPSC decay time. *P < 0.05, **P < 0.001. n.s., Not significant.

NTS NMDARs are required for some of the vagus-mediated effects of CCK on food intake (39, 40). In addition, our laboratory has shown that NMDARs help maintain fidelity of synaptic transmission at the ST-NTS synapse (26), demonstrating the necessity of NMDARs in NTS responses to evoked glutamate release. However, it is unknown how NTS NMDARs may contribute to charge transfer during spontaneous events (sEPSCs) in lepR neurons. To determine the contribution of NMDARs to sEPSC charge transfer, we performed voltage-clamp recordings under conditions that would allow NMDARs to participate and measured sEPSCs. Separate groups of lepR neurons were recorded in either 1.2 mM Mg^2+^ concentration ([Mg^2+^]) or 0 [Mg^2+^] aCSF, and all cells were confirmed to have connections to the solitary tract. When Mg^2+^ was omitted from the bath, sEPSCs had longer decay time constants (tau) (1.592 ± 0.221 vs. 3.165 ± 0.257 ms, *P* < 0.001) and transferred more charge per sEPSC (79.58 ± 11.06 vs. 147.43 ± 8.58 pA·ms, *P* < 0.001), whereas sEPSC current amplitude (32.54 ± 4.22 vs. 29.06 ± 2.27 pA, *P* = 0.587) and frequency (1.83 ± 0.32 vs. 1.60 ± 0.22 Hz, *P* = 0.234) were not significantly different (Fig. 5*A*). A cumulative fraction plot shows that the distribution of sEPSC decay times was shifted to significantly longer values (*Z* = 23.87, *P* < 0.001, K-S test; Fig. 5*A*). To confirm that NMDARs were responsible for the increase in sEPSC decay and charge transfer, we recorded in 0 [Mg^2+^] aCSF and included the NMDAR antagonist DCP. Bath application of 10 µm DCP reduced sEPSC tau (3.156 ± 0.24 vs. 1.989 ± 0.26 ms, *P* = 0.006) and charge transfer (135.76 ± 7.09 vs. 105.79 ± 6.82 pA·ms, *P* < 0.001) but did not significantly affect sEPSC amplitude (26.93 ± 1.41 vs. 26.01 ± 1.91 pA, *P* = 0.435) or frequency (1.34 ± 0.31 vs. 0.98 ± 0.27 Hz, *P* = 0.26; Fig. 5*B*). Moreover, a cumulative fraction plot shows that sEPSC decay times were shifted to significantly shorter values after DCP (*Z* = 8.604, *P* < 0.001, K-S test; Fig. 5*B*). These data indicate that NMDARs will contribute to spontaneous charge transfer in lepR neurons when Mg^2+^ is removed from the bath.

### NMDARs Contribute to CCK-Induced Firing in NTS lepR Neurons

**Figure 6.**
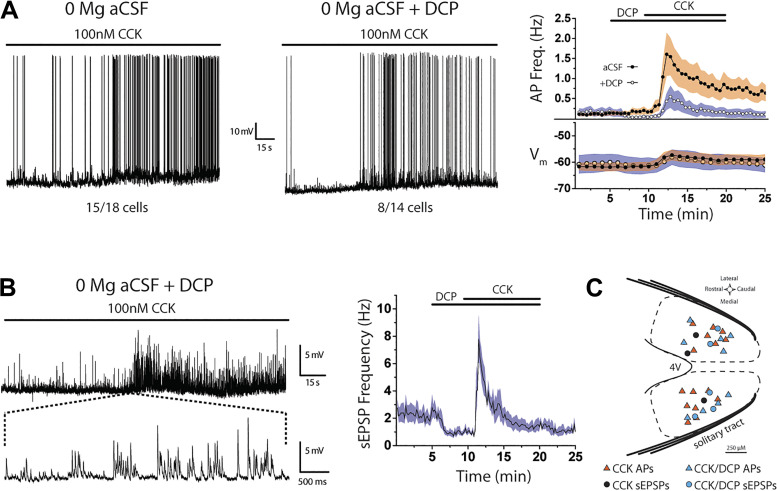
NMDA receptors (NMDARs) contribute to CCK-induced firing in nucleus of the solitary tract (NTS) leptin receptor-expressing (lepR) neurons. *A, left*: example current-clamp traces showing CCK-induced action potentials (APs) in 0 Mg^2+^ concentration ([Mg^2+^]) artificial cerebrospinal fluid (aCSF) ± D-4-[(2*E*)-3-phosphono-2-propenyl]-2-piperazine carboxylic acid (DCP). *Right*: time course of membrane potential (*V*_m_) and AP frequency during CCK application in 0 [Mg^2+^] aCSF (dark circles) and DCP (open circles). Time course is displayed as the average across cells (circles) ± SE (shaded regions). *B, left*: current-clamp trace showing CCK-increased spontaneous excitatory postsynaptic potential (sEPSP) frequency in the presence of DCP. *Right*: time course of sEPSP frequency during DCP and CCK application displayed as the average across cells (black line) ± SE (shaded region). *C*: distribution of recorded lepR neurons within the NTS. In 0 [Mg^2+^] aCSF, CCK stimulated APs (orange triangles, *n* = 15) or sEPSPs (dark circles, *n* = 3). After DCP pretreatment, CCK stimulated APs (blue triangles, *n* = 8) or sEPSPs (blue circles, *n* = 6).

Our results show that CCK increases spontaneous glutamate release onto lepR neurons, CCK-induced glutamate release is capable of stimulating action potentials, and NMDARs contribute to sEPSC charge transfer in the absence of Mg^2+^. We next examined whether removing Mg^2+^ would increase CCK-induced firing. Similar to Fig. 3, ST-EPSCs were confirmed in voltage clamp and then cells were allowed to reach a stable membrane potential in CC (*V*_m_ = −61.7 ± 1.6 mV; *n* = 18). Notably, there was no significant difference in resting membrane potential compared with CC recordings in 1.2 mM [Mg^2+^] (*P* = 0.063; see Fig. 4*A*). Bath application of 100 nM CCK induced AP firing in 15 of 18 neurons when Mg^2+^ was omitted from the aCSF [χ^2^(49, *n* = 18) = 254.83, *P* < 0.001, Friedman ANOVA; Fig. 6*A*], a markedly higher response rate compared with CC recordings in 1.2 mM [Mg^2+^]. AP frequency peaked 2.5 min into CCK treatment (1.61 ± 0.54 Hz) and then declined without reaching baseline levels. CCK-induced firing was also accompanied by significant membrane depolarization [χ^2^(24, *n* = 18) = 103.97, *p* < 0.001, Friedman ANOVA; Fig. 6*A*], which peaked 3 min into CCK treatment (+6.8 ± 0.8 mV) and remained elevated into the wash period. However, this depolarization was not statistically different from that seen after CCK in the 1.2 mM [Mg^2+^] condition.

To confirm that this increase in response rate and AP frequency was due to NMDARs, we repeated these 0 [Mg^2+^] experiments in a separate group of neurons and pretreated them with 10 µM DCP 5 min before coapplication of DCP and CCK. DCP pretreatment did not significantly reduce basal firing compared with 0 [Mg^2+^] control conditions [χ^2^(19, *n* = 14) = 29.32, *P* = 0.061, Friedman ANOVA; Fig. 6*A*]. Bath application of 100 nM CCK induced AP firing in 8 of 14 DCP-treated neurons [χ^2^(49, *n* = 14) = 132.84, *P* < 0.001, Friedman ANOVA; Fig. 6*A*] but did not cause a significant shift in membrane potential [*H*(24, *n* = 14) = 5.55, *P* = 1.0, Kruskal–Wallis ANOVA; Fig. 6*A*]. AP firing peaked 2.5 min into CCK/DCP treatment (0.55 ± 0.11 Hz) and returned to baseline levels by the end of the 5-min wash period. Importantly, comparisons of the final 15 min of recording between conditions show that CCK was less effective at stimulating APs in the presence of DCP [*F*(1,29) = 94.63, *P* < 0.001, 2-way ANOVA; Fig. 6*A*].

Finally, we measured sEPSP frequency in the six lepR neurons that did not fire action potentials in the presence of DCP + CCK. Interestingly, DCP alone decreased the frequency of sEPSPs [*F*(59) = 5.66, *P* < 0.001, 1-way ANOVA; Fig. 6*B*]. However, all six neurons showed a CCK-induced increase in sEPSP frequency [χ^2^(89, *n* = 6) = 220.95, *P* < 0.001, Friedman ANOVA; Fig. 6*B*], which peaked 1.5 min into CCK treatment (7.78 ± 1.74 Hz) and returned to baseline after wash. A map of recorded lepR neurons shows that CCK-induced AP firing and sEPSPs occurred in overlapping regions of the NTS (Fig. 6*C*).

### CCK-Induced Firing is Mediated by CCKR_1_

**Figure 7.**
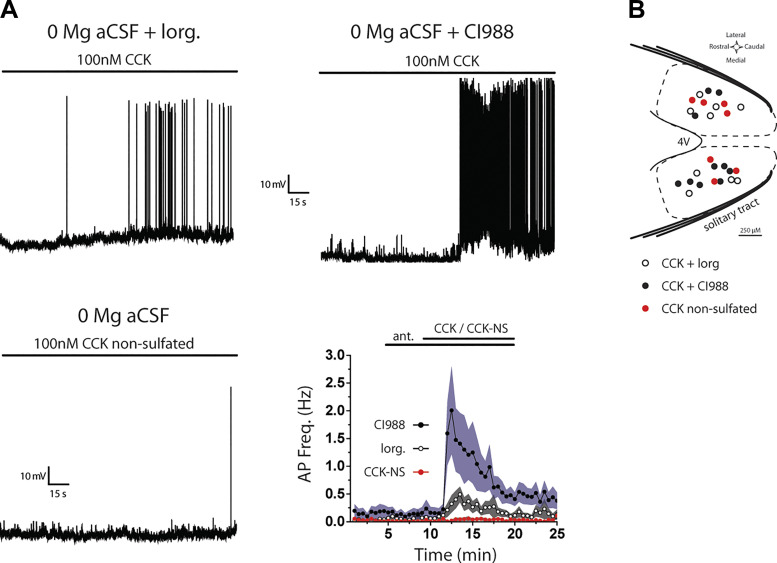
CCK-induced firing is mediated by CCK_1_ receptor (CCKR_1_). *A, top left*: current-clamp trace showing that 10 µM lorglumide attenuates CCK-induced firing. *Top right*: current-clamp trace showing that 100 nM CI988 does not block CCK-induced firing. *Bottom left*: current-clamp trace showing that 100 nM nonsulfated CCK (CCK-NS) does not stimulate action potential firing. *Bottom right*; time course of action potential (AP) frequency during lorglumide, CI988, CCK, and CCK-NS application displayed as the average across cells (dotted lines) ± SE (shaded regions). ant., the antagonists lorglumide (lorg) or CI988. *B*: distribution of recorded leptin receptor-expressing (lepR) neurons within the nucleus of the solitary tract (NTS). In 0 Mg^2+^ concentration ([Mg^2+^]) artificial cerebrospinal fluid (aCSF), lorglumide attenuated CCK-induced firing (open circles, *n* = 9), whereas CI988 did not block the effects of CCK on firing (black circles, *n* = 11). Bath application of CCK-NS alone did not stimulate firing (red circles, *n* = 7). 4V, 4th ventricle.

To determine whether the effects of CCK on lepR neuron firing in 0 [Mg^2+^] are mediated by activation of CCKR_1_, we repeated these CC recordings in the presence of a CCKR_2_ antagonist, CI988. Connection to the solitary tract was confirmed in voltage clamp, and lepR neurons were allowed several minutes to reach a stable membrane potential in CC before drug application (*V*_m_ = −60.1 ± 0.94 mV; *n* = 26). Pretreatment with 100 nM CI988 did not alter membrane potential [*F*(9) = 0.18, *P* = 0.996, 1-way ANOVA] or action potential firing [χ^2^(18, *n* = 10) = 20.48, *P* = 0.305, Friedman ANOVA; Fig. 7*A*]. Bath application of 100 nM CCK induced AP firing in all (10/10) CI988-treated neurons [χ^2^(18, *n* = 10) = 113.05, *P* < 0.001, Friedman ANOVA; Fig. 7*A*], and firing was followed by a small (+2.97 ± 0.7 mV) but significant membrane depolarization [χ^2^(9, *n* = 10) = 49.74, *P* < 0.001, Friedman ANOVA]. AP firing peaked ∼2.5 min into CCK/CI988 treatment (1.82 ± 0.77 Hz) and remained elevated for the remainder of the recording. Consistent with a lack of a CCKR_2_ effect, 100 nM nonsulfated CCK (CCK-NS) changed neither membrane potential [χ^2^(24, *n* = 7) = 113.05, *P* = 0.162, Friedman ANOVA] nor action potential firing [χ^2^(48, *n* = 7) = 50.99, *P* = 0.357, Friedman ANOVA; Fig. 7*A*], and firing remained low throughout the recording.

In contrast, pretreatment with a CCKR_1_ antagonist (lorglumide) blunted the effect of CCK. Ten micromolar lorglumide alone did not alter membrane potential [*F*(9) = 1.16, *P* = 0.334, 1-way ANOVA] or action potential firing [χ^2^(18, *n* = 9) = 17.94, *P* = 0.46, Friedman ANOVA; Fig. 7*A*] compared with control conditions. Interestingly, CCK was able to induce modest firing in the presence of lorglumide [χ^2^(18, *n* = 9) = 66.57, *P* < 0.001, Friedman ANOVA; Fig. 7*A*], and this firing was accompanied by a small (+2.04 ± 0.57 mV) but significant depolarization [χ^2^(9, *n* = 9) = 37.86, *P* < 0.001, Friedman ANOVA]. However, the peak effect of CCK on firing (0.49 ± 0.14 Hz) was significantly lower than that of either aCSF (see Fig. 6) or the presence of CI988, demonstrating that CCKR_1_ antagonism significantly attenuates the effects of CCK (Fig. 7*A*). A map of recorded lepR neurons shows that all drug treatment groups were recorded from overlapping regions of the NTS (Fig. 7*B*).

### Peripheral CCK Induces *c-fos* in NTS lepR Neurons

**Figure 8.**
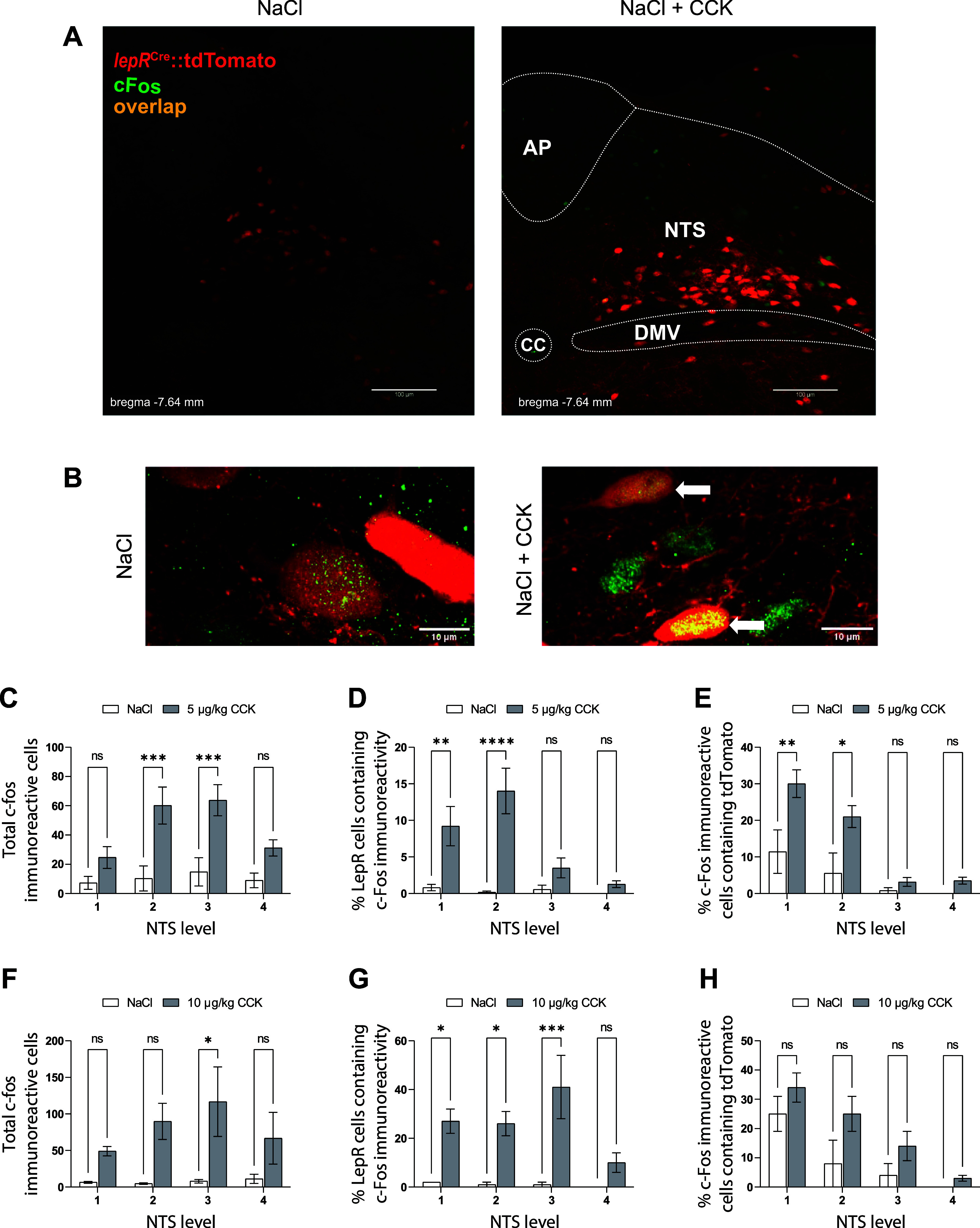
Peripheral CCK induces *c-fos* in nucleus of the solitary tract (NTS) leptin receptor-expressing (lepR) neurons. *A*: representative images of coronal hindbrain sections containing native tdTomato fluorescence (red) and stained to reveal c-fos immunoreactivity (green) after intraperitoneal injection of NaCl (*left*) or CCK (*right*). AP, area postrema; CC, central canal; DMV, dorsal motor nucleus of the vagus. *B*: ×63 magnification of NTS neurons with c-fos immunoreactivity (green) and/or native tdTomato fluorescence (red) after intraperitoneal injection of CCK. White arrows indicate colocalization. *C*: total numbers of c-fos-immunoreactive cells counted in coronal sections taken at 4 rostrocaudal levels of the NTS after injection of CCK (5 µg/kg ip). NTS *levels 1*, *2*, *3*, and *4* correspond to coronal sections taken at 7.92, 7.64, 7.48, and 7.20 mm caudal to bregma, respectively. *D*: % of total tdTomato-positive (lepR) cells counted in the NTS that colocalized with c-fos immunoreactivity after injection of CCK (5 µg/kg ip). *E*: % of total c-fos-immunoreactive cells counted in the NTS that colocalized with native tdTomato fluorescence after injection of CCK (5 µg/kg ip). *F*: total numbers of c-fos-immunoreactive cells counted in coronal sections taken at 4 rostrocaudal levels of the NTS after injection of CCK (10 µg/kg ip). *G*: % of total tdTomato-positive (lepR) cells counted in the NTS that colocalized with c-fos immunoreactivity following injection of CCK (10 µg/kg ip). *H*: % of total c-fos-immunoreactive cells counted in the NTS that colocalized with native tdTomato fluorescence after injection of CCK (10 µg/kg ip). Data are displayed as means ± SE; *n* = 6 mice per treatment for 5 µg/kg CCK, and *n* = 4 mice per treatment for 10 µg/kg CCK. *P < 0.05, **P < 0.01, ***P < 0.001, ****P < 0.0001 (by post hoc Šídák’s multiple comparison after 2-way ANOVA) ns, Not significant.

To determine whether peripheral CCK activates NTS lepR neurons in vivo, we examined the induction of *c-fos* expression following 5 µg/kg and 10 µg/kg injections of CCK. Intraperitoneal injection of 5 µg/kg CCK significantly increased *c-fos* immunoreactivity after 90 min compared with saline across four rostrocaudal levels of the NTS [*F*(1) = 31.43, *P* < 0.001, 2-way ANOVA; Fig. 8, *A* and *B*]. There was a significant main effect of NTS level on total *c-fos* expression [*F*(3) = 3.47, *P* = 0.025, 2-way ANOVA], with the highest *c-fos* induction occurring at NTS *level 2* (bregma −7.64 mm; NaCl 10.3 ± 8.5 cells per section, *n* = 6; CCK 5 μg/kg 60.2 ± 12.7 cells per section, *n* = 6; *P* < 0.001) and NTS *level 3* (bregma −7.48 mm; NaCl 14.8 ± 9.6 cells per section, *n* = 6; CCK 5 μg/kg 63.8 ± 10.6 cells per section, *n* = 6; *P* < 0.001; Fig. 8*B*). *c-fos* expression in NTS lepR neurons was extremely low after saline injections (< 0.5% colocalization), whereas CCK significantly induced *c-fos* in these cells [*F*(1) = 32.62, *P* < 0.001, 2-way ANOVA; Fig. 8*C*]. There was a significant main effect of NTS level on *c-fos* expression within lepR neurons [*F*(3) = 6.2, *P* = 0.0016, 2-way ANOVA], with the highest *c-fos* expression occurring at NTS *level 1* (bregma −7.92 mm; NaCl 0.81 ± 0.44%, *n* = 6; CCK 5 μg/kg 9.21 ± 2.67%, *n* = 6; *P* = 0.0026) and NTS *level 2* (bregma −7.64 mm; NaCl 0.17 ± 0.17%, *n* = 6; CCK 5 μg/kg 14.02 ± 3.11%, *n* = 6; *P* < 0.001; Fig. 8*C*). When representing colocalization as a percentage of the total *c-fos*-immunoreactive cells in the NTS, we found that a significant portion of *c-fos*-immunoreactive cells expressed lepR, with the highest percent colocalization occurring at NTS *level 1* (bregma −7.92 mm; NaCl 11.4 ± 5.9%, *n* = 6; CCK 5 μg/kg 30.0 ± 3.7%, *n* = 6; *P* = 0.0022) and NTS *level 2* (bregma −7.64 mm; NaCl 5.56 ± 5.56%, *n* = 6; CCK 5 μg/kg 21.03 ± 2.97%, *n* = 6; *P* = 0.0128; Fig. 8*D*). In a separate cohort of mice, we found that an intraperitoneal injection of a higher dose of CCK (10 µg/kg) produced a similar rostral-caudal pattern of *c-fos* expression across the NTS. Ten micrograms per kilogram significantly increased *c-fos* immunoreactivity after 90 min compared with saline across four rostral-caudal levels of the NTS [*F*(1) = 20.21, *P* < 0.001, 2-way ANOVA; Fig. 8*E*]. There was not a significant main effect of NTS level on total *c-fos* expression [*F*(3) = 0.807, *P* = 0.502, 2-way ANOVA; Fig. 8*E*]. *c-fos* expression in NTS lepR neurons was similarly low after saline injections (<0.5% colocalization), whereas CCK significantly induced *c-fos* in these cells [*F*(1) = 42.19, *P* < 0.001, 2-way ANOVA; Fig. 8*F*]. There was not a significant main effect of NTS level on *c-fos* expression within lepR neurons [*F*(3) = 2.91, *P* = 0.055, 2-way ANOVA; Fig. 8*F*]. Similar to the previous cohort, the highest percentage of *c-fos*-immunoreactive cells that colocalized with lepR occurred at NTS *level 1* (bregma −7.92 mm; NaCl 25.0 ± 6.0%, *n* = 4; CCK 10 μg/kg 34.0 ± 5.0%, *n* = 4) and NTS *level 2* (bregma −7.64 mm; NaCl 8.0 ± 8.0%, *n* = 4; CCK 10 μg/kg 25.0 ± 6.0%, *n* = 4; Fig. 8*G*). There were no effects of sex on total *c-fos* expression, percent colocalization within lepR cells, or percent colocalization within *c-fos*-immunoreactive cells, so data from both sexes were combined.

## DISCUSSION

Leptin has been shown to act on NTS neurons to control food intake (13, 16, 17, 21). NTS neurons that express lepRs receive both direct and indirect inputs from vagal afferent fibers in the solitary tract, suggesting they may integrate key information arriving from the periphery (21). Activation of NTS lepR neurons has been shown to inhibit food intake (22). However, nothing was known about the type and characteristics of the vagal afferent fibers that activate them. Here we report five new findings. First, NTS lepR neurons receive direct monosynaptic input from C-type vagal afferents as well as indirect, polysynaptic input from TRPV1-negative vagal afferents. Second, the vast majority of lepR neurons receive CCK-sensitive afferent inputs, and CCK-induced glutamate release is sufficient to stimulate action potential firing of lepR neurons. Third, postsynaptic NMDARs contribute to charge transfer during spontaneous glutamate release, and CCK-induced action potential firing is enhanced by NMDAR function. Fourth, the effects of CCK on lepR neuron action potential firing are mediated by CCKR_1_. Finally, peripheral (intraperitoneal) CCK induces in vivo *c-fos* expression in a subpopulation of NTS lepR neurons, presumably via vagal afferent activation.

### NTS lepR Neurons Receive Monosynaptic Input from C-Type Afferents and Polysynaptic Input from TRPV1-Negative Afferents

We previously have reported that NTS lepR neurons are activated by both monosynaptic and polysynaptic glutamatergic inputs after solitary tract (ST) stimulation (21). Here we show that most of the monosynaptic inputs are from C-type afferent fibers. Specifically, we found that the TRPV1 agonist capsaicin dramatically increased sEPSC frequencies. Furthermore, we observed that capsaicin attenuated ST-evoked EPSCs, likely due to a depletion in vagal afferent glutamate as has been shown for other NTS neurons that receive C-fiber input (28, 29, 49). Finally, we found that monosynaptic inputs to lepR neurons exhibit asynchronous glutamate release following ST stimulation. In the NTS, this form of glutamate release is a hallmark of direct input from TRPV1-expressing C-type vagal afferents (48). Asynchronous glutamate release is thought to be important for maintaining synaptic strength and fidelity of transmission at excitatory synapses (54–55) and has been shown to prolong postsynaptic action potential firing in NTS neurons (48). As TRPV1 channels are expressed almost exclusively by C fibers compared with other vagal afferent fibers, these combined observations suggest that the lepR neurons receive direct inputs from C fibers. Some studies have demonstrated that capsaicin also facilitates a glutamate-dependent increase in GABA release onto the dorsal motor nucleus of the vagus (DMV) of both rats and mice (57, 58). It is possible that capsaicin drives the activity of glutamatergic NTS neurons that have local connections with lepR neurons to increase glutamate release onto lepR neurons from nonafferent terminals that contribute to our observed responses. However, given the dramatic capsaicin-induced increase in sEPSC frequency and the findings that capsaicin both reduces the amplitude of direct ST-afferent inputs as well as increases asynchronous release within the same cells, this strongly supports the hypothesis that lepR neurons are directly activated by TRPV1-containing C fibers.

Although capsaicin treatment revealed significant monosynaptic input from C fibers, evoked ST-EPSCs were not completely abolished by capsaicin. Interestingly, the ST-EPSCs that remained had increased jitter and did not exhibit robust frequency dependent depression (FDD), suggesting that capsaicin-insensitive vagal afferent fibers make polysynaptic connections with lepR neurons. Significantly, a similar observation was made in another study of vagal afferents in mice (59), suggesting that it is not limited to lepR neurons. However, this pattern of innervation contrasts with that observed in rats, where ST-EPSCs in individual NTS neurons are either completely abolished after capsaicin treatment or are entirely unaffected, suggesting that A- and C-type fibers synapse on separate and distinct subpopulations of NTS neurons (28, 29, 60). Taken together, these results may suggest species differences in A and C fiber convergence. However, it is also possible that vagal afferents display different release properties or TRPV1 sensitivity between rats and mice, such that TRPV1 activation in mouse vagal afferents may be insufficient to completely block evoked glutamate release. Overall, however, our data suggest that NTS lepR neurons are monosynaptically connected to C-type afferents. C fibers are unmyelinated fibers that mediate reflex responses including those important for vagal afferent signaling from the GI tract (61–64), suggesting that lepR neurons can be activated by these signals. Interestingly, the fact that C fibers are associated with the expression of TRP channels, which can respond to physiological changes such as temperature (65–68), cannabinoids (69–72), and activation by kinases such as PKC (73, 74), means that lepR neurons could also be driven by vagal glutamate released by these stimuli.

### NTS lepR Neurons Receive Significant Input from CCK-Sensitive Fibers

The majority of the anorexigenic effects of peripheral CCK are mediated by C-type vagal afferent fibers (35). Here, we found that nearly all NTS lepR neurons (50/52 cells) were responsive to CCK, as defined by increases in sEPSC or sEPSP frequency and action potential firing. This result contrasts with the ∼45% of unlabeled NTS neurons that respond to CCK in rat brain slices (53). Moreover, the proportion of lepR neurons that responded to CCK in our preparation was higher than that for other mouse NTS neuron populations important for the control of food intake, such as in GLP-1-expressing [preproglucagon (PPG)] neurons (75) and catecholamine (CA) neurons (52). It is possible that the higher proportion of CCK-responding lepR neurons is related to the region of the NTS where these neurons are located (near the caudal end of the 4th ventricle, or obex), rather than being due only to neuronal phenotype. This interpretation is supported by the fact that the proportion of catecholamine neurons that respond to CCK also is higher in this region than in other NTS areas (Zhu, M and Appleyard, SM; unpublished observations in mice). In addition, responsiveness to CCK coincided with responsiveness to capsaicin, and CCK is believed to share or depend on TRP channel-mediated signaling pathways (76–78). Regardless, the exceptionally high number of NTS lepR neurons that are activated by CCK suggests that they are likely an important cell group to mediate its effects. In contrast, we found that serotonin rarely increased sEPSC frequency in lepR neurons, suggesting that they are not often downstream of serotonin-sensitive afferents. This is in contrast to catecholamine neurons, 90% of which are activated by serotonin-sensitive afferents (79).

Although our immunohistochemical studies confirm that *c-fos* is expressed in NTS lepR neurons after intraperitoneal CCK, the proportion of lepR neurons in which *c-fos* is induced is lower than the high response rate to CCK that we observed in the electrophysiological studies. There are several potential explanations for this. Animals were fasted overnight before CCK injections for the *c-fos* studies to remove the potential confounding effects of food intake; however, fasting can impair satiety responses to intraperitoneal CCK (80) and increases vagal expression of the inhibitory cannabinoid receptor 1 (81). Therefore, we may have observed a lower proportion of *c-fos*-immunoreactive lepR neurons than we would have with exogenous CCK or normal food intake. Moreover, it is also possible that *c-fos* is not induced in all cells that respond at the electrophysiological level to CCK, as the signal transduction pathways involved may be different. Furthermore, it is possible that local effects of CCK to increase lepR neuronal firing are stronger than the peripheral effects of CCK to drive vagal afferent activation of NTS neurons. Finally, our recordings may have been centered in a particularly CCK-responsive area of the NTS. Future studies could determine the topographical distribution of NTS lepR neuron responsiveness to CCK and other peptides, as well as whether fasting induces changes in the effects of either CCK or vagal stimulation on activation of lepR neurons.

### Identity of CCK-Sensitive NTS lepR Neurons

LepRs have been shown to colocalize with several neuropeptides and transmitters in mouse NTS neurons, including proopiomelanocortin (POMC), CCK, and PPG. However, in mice, lepR was not found to be colocalized in neurons expressing catecholamines, GABA, brain-derived neurotrophic factor (BDNF), neuropeptide Y (NPY), nesfatin, prolactin-releasing peptide, nitric oxide synthase (NOS), or cocaine- and amphetamine-regulated-transcript (CART) (82). Fifty percent of mouse NTS POMC-EGFP neurons [where POMC neurons are identified by the POMC promoter driving expression of enhanced green fluorescent protein (EGFP)] are activated by leptin at the level of phospho-signal transducer and activator of transcription 3 immunoreactivity (pSTAT3-IR), and these POMC-EGFP neurons represent ∼30% of all leptin-responsive neurons in the NTS (83). This level of colocalization is remarkably close to that observed by Garfield et al. (82), where 26% of mouse lepR-positive cells were also POMC-EGFP neurons. Mouse POMC-EGFP neurons are found in the medial NTS at the level of the area postrema, near the location of lepR neurons recorded in the present study (51, 82–85). POMC-EGFP neurons display electrophysiological properties similar to lepR neurons in the present study, as they are also fairly hyperpolarized (*V*_m_ ≈ −71 mV) and rarely fire at rest (51) Interestingly, all POMC-EGFP neurons show increased sEPSC frequency or moderate firing in response to CCK (51). Taken together, these data suggest that some of the lepR neurons recorded in this study could be POMC-EGFP neurons.

Almost two-thirds of PPG neurons have also been shown to express lepR in mice (82, 86). A loss of PPG does not, however, meaningfully impact the inhibition of food intake following activation of lepR neurons (22). Interestingly, the electrophysiological properties of PPG neurons differ from our observations of the lepR neurons. Most NTS PPG neurons sit at depolarized resting membrane potentials compared with lepR neurons in the present study, and PPG neurons also exhibit spontaneous or burst-firing activity, with <9% being totally quiescent (87). In addition, only half of PPG neurons respond to CCK, and this effect is thought to be indirect (i.e., polysynaptic) through activation of catecholamine neurons (75). It is conceivable that our recordings were from a distinct subpopulation of PPG neurons located in the medial-caudal NTS and that these neurons receive direct input from CCK-sensitive fibers, but this hypothesis remains to be tested.

Approximately 44% of NTS CCK-expressing neurons also express lepR (82). Recent studies show that these neurons are sensitive to nutritional state, and chemogenic and optogenetic activation of these cells inhibits food intake (88–90). However, no studies to date have characterized the electrophysiological profile of these cells for comparison with NTS lepR neurons. Interestingly, however, activation of NTS POMC (91), PPG (92–94), and CCK (88–90) neurons all result in inhibition of food intake, consistent with the proposed role of lepR neurons in the NTS (13, 17, 18, 20, 22, 95).

There may be interesting species differences in the expression of lepRs between mice and rats, as lepRs are expressed in PPG neurons in mice but not rats (96). However, there are also similarities, as NMDARs appear to be critical for the effects of leptin in both mice and rats (21). In the future, it will be interesting to compare what drives lepR-expressing neurons in both animal models. However, currently there is no readily feasible way of identifying the lepR neurons for electrophysiological recordings in rats. One potential caveat of using LepR-Cre mice to identify the lepR-expressing neurons is that Cre recombination could occur at any time, and neurons will be labeled even if the receptor was only transiently expressed during development, as has been reported for other Cre lines (85). Indeed, Garfield et al. (82), using the same LepRb-Ires-Cre::tdTOM mouse line as well as a LepRCre-EYFP mouse line, found that the tdTomato- and GFP-immunoreactive neurons in the rostral NTS did not completely colocalize with leptin-induced phosphorylated signal transducer and activator of transcription 3 (pSTAT3) (76). However, leptin did induce pSTAT3 in ∼70–80% of the LepR-Cre neurons in the caudal NTS, which encompasses the region we are recording from in this study. Furthermore, leptin may not activate pSTAT3 in all lepR+ neurons, as leptin activates CREB and pErk in the hippocampus but not pSTAT3 (97) and pSTAT3 could be indirectly activated. However, as >95% of lepR neurons in this study responded to CCK (50/52) and all responded to capsaicin, we believe that our results can be extrapolated to lepR neurons in the caudal NTS of adult mice.

### NMDARs Increase Sensitivity of NTS lepR Neurons to “Spontaneous” Glutamate Release from Vagal Afferents

Previous studies from our laboratory revealed that NMDARs are important for NTS neuronal responses to vagal afferent glutamate release, in terms of both ST-evoked EPSCs and action potential firing (26). In particular, we found that NMDARs prolong activation of NTS neurons by high-frequency vagal firing, such as occurs after a meal or CCK-induced activation of the vagus (98). In addition to evoked glutamate release, we (51, 79, 99) and others (53, 100) have found that vagal afferents often release significant amounts of glutamate spontaneously and that increased spontaneous release can drive NTS neuronal firing. Here, we found that NMDARs contribute to NTS neuronal responses to spontaneous glutamate release and that activation of NMDARs leads to greater sEPSC charge transfer. More specifically, NMDAR activation increased sEPSC current decay times rather than current amplitudes, suggesting that NMDAR activation extends the time window for individual sEPSCs to summate into action potentials. These observations are similar to what is seen in other brain regions (101–103) and suggest that activation of NMDARs by spontaneous glutamate release may increase glutamate’s ability to drive lepR neuronal firing independent of vagal activation. Indeed, we have demonstrated that the response rate, maximal effect, and duration of CCK-induced firing are all at least twofold greater when NMDARs are allowed to contribute to spontaneous glutamatergic transmission. Importantly, most CNS NMDARs are blocked by Mg^2+^ at negative membrane potentials (104, 105), and it is often necessary to hold NTS neurons at positive potentials or record under Mg^2+^-free conditions to study NMDAR currents (106–110). This suggests that local synaptic or somatic depolarization is required before NMDARs can contribute to sEPSCs. Taken together, it is possible that lepR neuron NMDARs participate in vagus-NTS synaptic transmission only during periods of heightened vagal activity, such as increased vagal firing in response to satiation signals like CCK (111, 112), or during times that the lepR NTS neurons receive excitatory inputs from other brain regions or neighboring NTS neurons at the same time, allowing the NMDARs to act as coincidence detectors (113) to increase the sensitivity of the lepR neurons.

### Effects of CCK Are Mediated by CCKR_1_ Receptors

The CCKR_1_ antagonist lorglumide has been shown to block CCK-induced glutamate release from vagal afferents and subsequent action potential firing in NTS neurons (51, 53). Here, we found that lorglumide significantly attenuated CCK-induced firing in the lepR neurons. CCKR_2_ do not appear to contribute to CCK-induced activation of lepR neurons since nonsulfated CCK, which preferentially activates CCKR_2_, did not trigger EPSPs or otherwise alter lepR neuron activity. Moreover, the CCKR_2_ antagonist CI988 failed to attenuate CCK-induced firing. Interestingly, lorglumide alone did not alter lepR neuron firing or resting membrane potential, suggesting that there is little to no constitutive activity of CCKR_1_ or central CCK “tone” onto lepR neurons in ex vivo NTS slices. However, this horizontal slice preparation may have cut inputs that drive the activity of CCK neurons, and future studies should examine the effects of endogenous CCK. Together, these results are consistent with reports indicating that the NTS neurons receive input from CCKR_1_-expressing vagal afferents (114–116).

### Physiological Implications

Stimulation of lepR neurons in vivo inhibits food intake (22). Our results demonstrate that the activity of these neurons can be driven by CCK-sensitive vagal C fibers, which are known to carry critical GI information to the brain after a meal. We also found that peripheral injection of exogenous CCK induces *c-fos* in lepR NTS neurons and that these neurons are particularly sensitive to the concentration of CCK we used. Interestingly, both fats and proteins have been shown to be particularly effective at releasing CCK in the periphery and activating CCK-sensitive afferents, suggesting that lepR neurons may be particularly responsive to these nutrients (5, 117–119). We also found that lepR neurons receive multiple afferent inputs, both direct and indirect, which again suggests that they could receive multiple types of information. Interestingly, lepR-expressing NTS neurons have been shown to be in close opposition with afferents from the carotid artery, suggesting that they may integrate information across multiple modalities of information (120).

Previous studies have demonstrated cooperative actions of CCK and leptin along the vagus-NTS neuroaxis. Peripherally, leptin and CCK have additive effects on vagal afferent firing (98, 121) and both activate cell bodies of vagal afferent neurons innervating the stomach and duodenum (15, 27, 122). Centrally, intracerebroventricular (ICV) leptin injections increase the ability of peripheral CCK to reduce food intake and body weight (123, 124). However, it was unclear how CCK and leptin interact at the level of the NTS. Results from the present study suggest that the vagus-NTS synapse is an important point of convergence for the effects of CCK and centrally acting leptin. In support of this idea, we have shown that NTS lepR neurons receive significant input from CCK-sensitive fibers and that CCK-induced glutamate release is sufficient to drive lepR neuron firing. We have previously shown that leptin potentiates NMDAR-mediated currents, leading to increased ST-NTS synaptic throughput (21), suggesting that centrally acting leptin would increase the sensitivity of NTS neurons to CCK activation of vagal firing.

We found that CCK-induced glutamate release is sufficient to drive neuronal firing in an ex vivo brain slice preparation. This raises the interesting possibility that NTS sources of CCK could activate vagal afferent terminals to drive neuronal firing independent of the peripheral vagus. Stimulation of NTS CCK neurons inhibits food intake via their projections to the paraventricular nucleus of the hypothalamus (88, 90) and the parabrachial nucleus (89, 90). One intriguing question is whether these NTS CCK neurons release CCK locally. This is an important question since local release of CCK could impact the activity of multiple NTS cell groups involved in the control of food intake, including the lepR neurons (shown here) as well as PPG (75, 125), catecholamine (52, 126), and POMC-EGFP (51, 127) neurons.

### Perspectives and Significance

This study is the first to characterize what types of vagal afferents directly excite leptin receptor-expressing (lepR) neurons in the nucleus of the solitary tract (NTS). We found that NTS lepR neurons are strongly and directly activated by monosynaptic input from cholecystokinin receptor 1 (CCKR_1_)-expressing vagal afferent C fibers. As we have previously shown that leptin can potentiate the effects of incoming afferents to activate these neurons, this may provide a key mechanism by which leptin can potentiate the effects of CCK on NTS activation. Moreover, we found that the excitatory effects of CCK on action potential firing are much greater under conditions when NMDARs contribute to spontaneous synaptic transmission, and NMDARs are known targets for leptin-induced potentiation and plasticity (128–132). Taken together, it is possible that NMDAR-mediated signaling may underlie some of the cooperative effects of CCK and leptin on brain stem neurons and contribute to the anorexigenic properties of these peptides. In addition, we found that central CCK is sufficient to drive the firing of the lepR-expressing NTS neurons, and this could be a mechanism by which their activity is increased, independent of vagal firing. As activation of lepR neurons results in inhibition of food intake, this could represent a central mechanism that could drive inhibition of food intake, without ingestion of a meal.

## DATA AVAILABILITY

Data will be made available upon reasonable request.

## GRANTS

This work was funded by National Institutes of Health Grants DK083452 (to S.M.A.) and DK052849 (to R.C.R. and S.M.A.).

## DISCLOSURES

No conflicts of interest, financial or otherwise, are declared by the authors.

## AUTHOR CONTRIBUTIONS

D.M.N., R.C.R., and S.M.A. conceived and designed research; D.M.N., L.B., R.C., and E.T.W. performed experiments; D.M.N., L.B., R.C., and E.T.W. analyzed data; D.M.N., L.B., R.C., E.T.W., R.C.R., and S.M.A. interpreted results of experiments; D.M.N. and E.T.W. prepared figures; D.M.N. drafted manuscript; D.M.N., R.C., E.T.W., R.C.R., and S.M.A. edited and revised manuscript; D.M.N., L.B., R.C., E.T.W., R.C.R., and S.M.A. approved final version of manuscript.
